# Dyadic Interviews versus In-Depth Individual Interviews in Exploring Food Choices of Norwegian Older Adults: A Comparison of Two Qualitative Methods

**DOI:** 10.3390/foods10061199

**Published:** 2021-05-26

**Authors:** Fifi Kvalsvik, Torvald Øgaard

**Affiliations:** The Norwegian School of Hotel Management, University of Stavanger, 4021 Stavanger, Norway; torvald.ogaard@uis.no

**Keywords:** dyadic, in-depth individual, interviews, qualitative method, older adult, food choices

## Abstract

The term “dyadic interview” refers to interviewing two participants together. Although there has been an increase in the use of dyadic interviews as a data collection method in qualitative studies, the literature on the use of this method with older adults is limited. This study was designed to explore the suitability of dyadic interviews as a method of data collection among older adults living at home. The study involved a direct comparison of the data obtained from dyadic interviews and in-depth individual interviews concerning older adults’ food choices. The study sample consisted of eight dyads for the dyadic interviews and six participants for the in-depth individual interviews. The dyads were composed of pairs who share a pre-existing relationship as well as pairs of strangers. We also discussed the role of participant selection and pairing in dyadic interviewing and how the interactions between the dyads may affect the result. Our results indicated that dyadic interviews can be used as an important data collection tool for home-living older adults, particularly when exploring a topic that often involves a dyadic decision. Our findings can be useful for researchers to make a more informed choice when choosing qualitative data collection methods, particularly when interviewing older people.

## 1. Introduction

As a result of demographic changes, research involving older adults is becoming more important. While research carried out on older adults is feasible, it also presents many challenges. Features of old age, such as physical and cognitive characteristics, often influence the process and outcome of interview data [1,2] and can pose a threat to the validity of a study [1,2].

Interviews are typically seen as the “gold standard” in qualitative research [3]. Today, the most commonly used interviewing methods are in-depth individual and focus group interviews [4,5]. In reality, these two methods seem to be the default choice when setting up qualitative studies.

Due to the unique characteristics of an older population, special attention should be paid to data collection methodologies [6]. Even though there is comprehensive literature on how to conduct interviews with older adults [7], little is known about the relative merits of alternative data collection methods for older adults [8]. Therefore, research exploring data collection methods beyond the simple traditional approach is needed.

Multiple authors have discussed the potential advantage of dyadic interviews; for example, dyadic interviews allow participants to stimulate ideas that might not have been either recognized or remembered [9,10]. A number of studies employing dyadic interviews have been published in health studies and family research. However, little attention has been paid to the dyadic interview as an alternative data collection method for older adults. Hence, more research is needed to yield structured knowledge of the merits of dyadic interviews as a data collection method with older participants. Our study seeks to fill this knowledge gap by empirically comparing dyadic interviews with the most commonly used method, which is in-depth individual interviews.

Aside from the above consideration of the sample characteristics’, we took the nature of the research topic into consideration. Food choice involves an isolated choice, but what a person decides to eat and how they arrive at a decision is often a form of collaborative decision with others in everyday life, for example, significant others, family members, or work [11]. It has been suggested that the use of dyadic interviews in exploring research topics related to collaboration can contribute to the co-creation of new knowledge [12].

The aim of this study is to explore whether a dyadic interview is a viable method for collecting data from home-living older adults when exploring their food choices. This study draws on a subset of data collected as part of an exploratory investigation into home-living older adult’s food choices. 

The study was not set out to establish the optimum research method. Instead, we are interested in comparing the ability of the two methods to elicit information in the context of food choice and analyzing each method’s benefits and drawbacks. To the best of our knowledge, there are no prior studies reporting findings on food choice with older adults as a sample that uses the dyadic interview as a data collection method.

## 2. Background

The growth of the older population worldwide is inevitable. There are about one billion people aged 60 and over today, and this will double by 2050 [13]. This rapid growth of the older population will have profound implications for each of us and the communities we live in [14].

Much has been written about how the aging population will put a strain on public finances and the welfare system. The main concern is the increasing demands placed on the healthcare system to care for this aging section of the population. 

It is common knowledge that most older adults prefer to continue living independently in their own homes [15]. For example, more than 75 percent of older adults in Australia, New Zealand, Europe, and Northern America live independently at home [16]. Having older adults remain in their own homes for as long as possible has a positive effect on public finances, welfare systems, and older people themselves [17].

Most older adults can continue to live independently in their own homes as long as they stay healthy. An important factor in improving healthy aging is adequate food and a nutritionally sound diet [18,19]. Thus, the role of food in maintaining health in older adults living at home is an important area of concern in today’s research.

Older adults may view food, nutrition, and health very differently from experts in food and nutrition [20]. Understanding how older adults choose foods and conceptualize a healthy diet offers important perspectives that can inform public policy makers and practitioners to support home-living older adults in healthy aging through their food intake.

The research in this area has shown that multiple factors influence why older adults choose one food type over another [21,22]. In this study, we will use a framework proposed by Host, McMahon, Walton and Charlton [22] to evaluate the attribute variability between the dyadic and in-depth individual methods. Based on this framework, three domains influence the food choice of older adults. These domains are the changes associated with aging, psychosocial aspects, and personal resources. 

The changes associated with aging refers to the physiological changes related to age, including taste, poor dentition, loss of appetite, mobility or functional limitations, and illness or medical conditions. The psychosocial aspects refer to life course, living arrangement, self-perception of health status, desire for independence, lack of motivation or energy, and interest in health or nutrition. Personal resources are listed as transportation, income, personal support, food preparation skills, access to quality products, and dietary resilience to overcome barriers. 

As described above, food choice is a multi-faceted phenomenon; therefore, we chose two interview methods to explore and shed light on this phenomenon. In the section below, we will present the two data collection approaches.

### 2.1. Data Collection Approaches

For this exploratory study, we chose to apply two data collection approaches, the dyadic [9] and in-depth individual [23] interview.

The first approach is the dyadic interview [9]. The term dyadic interview refers to interviewing two participants together to collect useful data for a research project [9]. Although the dyadic interview has appeared in studies since the 1970s under the label “joint interview” [24], the current literature on the dyadic interview is still fragmented and incomplete. Typically, in dyadic interviews, a researcher is primarily interested in the interaction between the two participants because the interaction in the dyadic interview is what produces the data [25]. 

Dyadic interviews commonly involve two participants that share a pre-existing relationship, such as married couples and caregivers–patient relationships [10,26]. Very little has been written to date about dyadic interviewing with pairs of strangers [9]. There is, however, reason to believe that a similar result may be yielded from strangers who share a common experience [27]. We, therefore, decided to include stranger pairs in this study.

Prior research has described the unique advantages of the dyadic interview as a tool for collecting data from a specific group of people, for example, people with early-stage dementia. It has been suggested that people with dementia are often overwhelmed when facing new groups of people. Dyadic interviews can eliminate this drawback by carefully pairing two participants, which promotes a sense of safety [9]. Dyadic interviews also allow participants to have more time to process what has been said and to formulate their responses. [9]. This claim, however, has not been tested empirically with older adults living at home.

We chose the dyadic interview method for three reasons. First, it allows the researchers to observe interactions between the pair of interests [24]. Second, dyadic interviews allow the content to be extended beyond what might have been possible in individual interviews [25].

Third, the dyadic interview is well suited to eliciting knowledge from individuals who need additional time to process or recall information [9]. The literature shows that changes in cognition often occur with normal aging [8]. Thus, dyadic interviews allow researchers to explore older adults’ perspectives while accounting for the older population’s unique characteristics.

While there are many advantages, there are some drawbacks to using the dyadic interview. One potential drawback is the problem of domination [24]. Within a dyad, one person might dominate the interview by constantly talking and dismissing the other participant [24,28]. Other drawbacks reported are potential conflicts triggered within a dyad [29] and acquiescence bias in a relationship where power is not distributed equally within a dyad [30].

In the in-depth individual interview, the second approach, the dynamic within the interview is fundamentally different. The in-depth individual interview typically involves one on one interaction between a participant and a researcher [23]. Unlike the dyadic interview, the interaction in the in-depth individual interview is a way of building rapport between the participant and the researcher rather than being part of the data itself [25]. This approach requires a researcher to engage with a participant in seeking “deep” information and knowledge [31]. 

The in-depth individual interview was selected because it represents the most widely used data collection method in qualitative studies [5]. In addition, the in-depth individual interview permits and encourages the participant to tell his or her own story, which allows researchers to explore a phenomenon from an individualistic perspective [32]. Therefore, this method enables us to grasp and articulate individual participants’ multiple views [23].

In-depth individual and dyadic interviews each have their own merits and drawbacks. Nevertheless, it has often been argued that individual interviews tend to reveal more detailed information than other methods [33].

### 2.2. Comparison Framework

To compare the two data collection methods, we draw on four criteria from Steenkamp and Van Trijp [34]. These criteria are the number of attributes elicited, the variety of attributes elicited, the efficiency of data collection, and the participants’ reactions to elicitation methods. 

The purpose of attribute elicitation is to uncover attributes; a data collection method that elicits more attributes may, therefore, be considered better [35]. However, the attributes need to represent unique pieces of information [35]. This leads to our first research question, which is as follows:

RQ1: Which methods generate a higher number of unique attributes?

In addition to the number of attributes, we focus on the type of information the methods produce. This shapes our second research question, which is as follows:

RQ2a: Do the methods produce different types of information and, if so, what types of information?

RQ2b: Which attributes and how many are captured based on the three domains for the determinants of food choice in older adults [22]?

From a practical standpoint, the ability to effectively collect and analyze data is increasingly important, especially in a study where speed and cost are a priority, for example, marketing research [34]. This leads to our third question, which is as follows:

RQ3: How demanding is each data collection method in terms of the time needed to collect and analyze the data?

One other key aspect to consider in attribute elicitation is the participant. A failure to give consideration to the participants may reduce the accuracy of the responses [34], which results in a less valid response [36]. This leads to the final research questions, which are as follows:

RQ4: How suitable are the applied methods for the target sample?

RQ5: How do participants respond to and perceive the interview methods?

## 3. Materials and Methods

### 3.1. Participants

The term “older adults” has been defined differently in the literature. One way of measuring old age is using a fixed chronological age without regarding how healthy a person is, how a person functions, or whether a person is actively working or retired [37]. For the purpose of this study, we have defined an older adult as a person aged 60 and over. Although we agree that chronological age is not the best predictor variable, it is the most common way to measure age [38]. 

With that said, we also consider “older adults” based on their characteristics, for example, health and physical strength [37]. In this respect, one wheelchair user (58 years old) who is a member of the senior activity center was included in the study sample. 

What follows is the nature of the research topic. Food choice is a complex construct that often involves other people connected to us [39]. To examine food choice as a construct and its variation, we also included younger participants in our parent–child dyad.

### 3.2. Interview Design 

For the purpose of this study, we used the general interview guide approach [40] to gather data from all the participants. With this approach, we can ask or change questions based on participant responses to previous questions [40]. Both the dyadic and in-depth individual interviews were identical in terms of the topics and questions. The purpose of making the topics and questions identical was to ensure the comparability of the data [41,42].

In terms of the questions’ sequence, we organized the interview questions by following the “funnel” format [42], moving from broad to specific areas. Furthermore, the types of questions asked were based on the guidelines proposed by Billups [43]. These questions include, for example, (a) what a person is doing/has done, (b) what a person thinks, (c) establishing the facts, and (d) how they feel [43]. With this as a guideline, the participants were instructed to describe (1) their daily eating practices, (2) their knowledge of a healthy diet, (3) identification factors in an older person’s life that can affect food choices, and (4) self-efficacy in dietary behavior.

### 3.3. Sampling Method

Our initial recruitment strategy entailed placing flyers in the mailboxes of senior housing complexes. The gatekeepers were informed of the study, and interested participants were instructed to contact the researchers by phone. We were, however, unable to recruit enough participants within the expected time frame using only this strategy. To overcome this issue, we adopted a more proactive recruitment strategy, in which we recruited participants from senior activity centers in the district. This approach involved a 30-minute presentation of our project to the members of activity centers. Subsequently, those interested in participating were asked to set up a time and place for an interview. As time progressed, we employed street-intercept recruitment strategies in public places, such as public libraries, coffee shops, and shopping centers, to increase the number of participants. Those who agreed to participate were given the option of an individual interview or to be paired up with someone else.

To gain as broad understanding as possible of older adults’ food choices and perceptions of a healthy diet, we deliberately sampled for heterogeneity [44]. We chose a sample of older adults who varied in age, gender, occupation, employment status, marital status, and living situation. In addition, for the dyadic interviews, the participants were paired together based on different types of relationships (see Table 1). We expected the variety of participants to enable us to capture varying perspectives on the phenomenon being studied [45].

As a result, we recruited 22 participants. Of those, 16 completed a dyadic interview and 6 completed an in-depth individual interview. Of the 16 participants in the dyadic group, the following 8 dyads were established: 2 married couple dyads, 2 parent–child dyads, 2 friend dyads, and 2 stranger dyads.

The participants’ characteristics are presented in Table 1 and Table 2.

### 3.4. Data Collection

The data collection was carried out in a district of western Norway. The study was reviewed and approved by the Norwegian Centre for Research Data (2019/502106), and written informed consent was obtained from all the participants individually before the data collection was initiated. The interviews were held at a time and place of the participants’ choice, including participants’ homes, cafeterias at senior centers, libraries, and coffee shops. 

#### 3.4.1. Dyadic Interview Implementation 

Each dyadic interview started with the participants introducing themselves and continued with a discussion of their favorite food. This eventually progressed into a dialogue around food choices and healthy diets. Each interview session lasted between 50 min and 1.5 h. Once the interview started flowing like a conversation, we focused on observation and taking field notes. This, however, was not the case with all the dyads in the study; some required more probing to keep the conversation flowing. All the interviews were audio-taped and subsequently transcribed verbatim. 

#### 3.4.2. In-Depth Individual Implementation

Participants in the in-depth individual interview started with the same study protocol as the dyadic interview did. After introducing themselves, participants were asked to describe their favorite food, “Tell me about your favorite food”. We continued the interview by asking participants open-ended questions and, depending on their response, we continued with questions that sought to obtain clarification. Each interview lasted between 40 min and 1 h. Field notes were taken while conducting the interviews. All the interviews were audio-taped and subsequently transcribed verbatim. 

### 3.5. Data Analysis

Our data analysis of the studies consisted of three phases. 

First, we conducted a within-study content analysis for the dataset produced using each data collection method. Content analysis examines data in order to understand what it means to people [46]. Upon completion of each interview, the audio files were listened to several times, and verbatim transcriptions were prepared for each interview. To ensure the accuracy of the transcriptions, the same datasets were transcribed by a transcriber who was not involved in the data collection.

In an attempt to become familiar with the “voices” of participants, the transcript was read thoroughly several times by one or two authors, after which the units of meaning were identified. These units were then abstracted and labeled with a code separately by each author. Any disagreement in the code description was resolved through discussions among the authors during project meetings. 

The coding process was iterative, and the categories evolved as the analyses progressed. After careful analysis, the codes were then grouped into categories and subcategories [47].

In the second round of the analysis, we evaluated differences in the types of information elicited between the two data collection methods. We followed the deductive content analysis approach [47], where attributes were assessed based on the three key domains proposed by Host, McMahon, Walton and Charlton [22].

Third, a cross-study analysis was conducted to compare the responses elicited in the dyadic interviews and the in-depth individual interviews. The comparison of the two elicitation methods was divided into two parts. The first part included the result for the information elicited (number of attributes elicited and attribute variability), while the second part contained the result for the procedural dimensions (efficiency in data collection and participants’ feedback). 

The number of attributes elicited was established through a simple count of the attributes elicited in each interview. The attribute variability was analyzed based on the type of information that was elicited across the two methods. With regard to the efficiency of data collection, we took the time spent on conducting the interviews, transcription, and analyzing the data into consideration. For the participants’ feedback on the methods applied, we ascertained this by asking the following questions after each interview: “How do you feel about our discussion?”, “How do you feel about this interview?”, “Do you have any other comments?”

### 3.6. Trustworthiness of Data

To verify the accuracy and trustworthiness of the present study, we used the criteria established by Lincoln and Guba [48], as follows: credibility, transferability, dependability, and confirmability. Credibility was achieved through prolonged engagement with the participants, field note writing, and the use of triangulation. For this study, we used the following three types of triangulation: (1) method triangulation, (2) investigator triangulation, and (3) data source triangulation [49,50].

With regard to transferability, the participants’ demographics and context were described in detail to allow the reader to decide whether the result was transferable. Furthermore, to improve the dependability and confirmability, a detailed description of the research procedure was provided, allowing others to conduct follow-up studies.

## 4. Results

The results are divided into three sections. We first present how each method elicited attributes within the three key domains that determine food choice (the changes associated with aging, psychosocial aspects, and personal resources). Comparisons are then made between the dyadic interviews and the in-depth individual interviews. Finally, we focus on the merits and drawbacks of each method.

### 4.1. Dyadic Interview

The dyadic interviews generated a rich and broad range of data, as it facilitated the participants to share their perspectives and experiences and allowed for comparisons to be made with the other participant in the dyad. Participants’ clarifications of their food choices and perceptions of a healthy diet in the discussion covered gender perspectives on food, food politics, and self-construal. 

Furthermore, this approach allowed us to elicit factors within the three key domains identified as influencing home-living older adults in relation to food choices and a healthy diet.

When asked about everyday food choices, some participants reported that they had changed their diet because of health-related issues, while others mentioned that their diet had remained the same. This response implies that changes associated with the aging domain can be captured using the dyadic interview method with home-living older adults. An example is offered by the following comment:

Wife: “We mostly eat seafood, things that come from the ocean. In a period of our life, we have a very different diet; we always have meat in the freezer (looking at her husband), meat from the wild from hunting”.

Husband: “Yes, we have an issue with our stomach, so we go a bit from meat and eat more seafood. We have a cabin next to the ocean, so we eat more fish. In addition, diabetes in the family, so we need to be careful with sweet things”. (Married couple 2).

The dyadic interviews also captured psychosocial aspects, since all participants reported that changes in their food choices were affected by life stages, for example, family formation, children moving away, and spousal negotiations around food choice. A few participants also attributed the changes in their food intake to living alone after separation or the passing of a spouse. Despite this, all the participants reported feeling positive about their health, and few had a personal interest in food and nutrition.

Father: “My diet is a bit different now that I live alone. I am divorced. Before I eat a lot of pasta and salad. My ex-wife cook, so I eat whatever she puts on the table (laugh).”

Son: “My mom, she loves pasta. For me, I eat differently when my girlfriend is here; she is vegetarian. I am not vegetarian when she is not here (laugh).”

Father: “I often eat green vegetables with my girlfriend.” (Father–son).

Regarding the domain of personal resources, the participants described family members and friends as the primary source of support in food-related activities. Furthermore, the participants generally believed that they have adequate nutrition-related knowledge and are aware that certain foods are associated with a healthier diet. An example is offered by the following comment: 

Mother: “No sweet things, I need to be careful with milk, no lamb ribs. Lots of vegetables and fruit is good for me.”

Daughter: “I’m very focused on getting enough nutritious things in me…that’s the reason I don’t take any supplements, rather the thing that I get it naturally from food, for example, the green in the green vegetable, like spinach, something like that iron.” (Mother–daughter).

This articulation suggests that dyadic interviews can be used to capture the domain of personal resources in older adults living at home.

### 4.2. In-Depth Individual Interview

The in-depth individual interviews captured an overall picture of older adults’ attitudes towards healthy diet behavior and their food choice determinants. The participants reflected on their food choices and diet through their own experiences by describing past and present experiences related to food and a healthy diet in a home setting. 

The in-depth individual interview method captured the full range of changes associated with the aging domain. All of the participants reported that they experience one or more challenges related to maintaining a “healthy” diet, such as compromised senses (taste and/or smell), a reduced appetite, poor dentition, and digestive conditions. Additionally, two of the participants reported problems with mobility. Consequently, the participants chose to stick to their current diet. They felt that their everyday diet worked well for them and, most importantly, was manageable.

“I am not sure, but I think it started when I started going to the senior activity center. I have diarrhea every time I come home. I thought maybe is the bread, so I stop eating that. I then started to eat different bread. It was better. I know it is not the food there; it is my stomach. I can’t drink some of the juices in the store too; it is too strong for my stomach, so it starts hurting.” (P3).

Concerning psychosocial aspects, participants reported that living alone after the loss of a spouse had a negative effect on their food intake. The examples given included not having a motivation to prepare food for one person, only eating pre-prepared food from a store, and skipping meals. Of these participants, one did actually live with a spouse but stated that they did not have any interest or the energy to prepare food. Despite the changes in food consumption, all the participants believed that their diet was healthy enough. This result suggests that in-depth individual interviews are well-suited for capturing the psychosocial aspects of older adults’ food choices.

“When my wife was still alive, it was me who makes dinner sometimes. I like making dinner because she appreciates it, but now, nah… (thinking), I don’t want to stand in the kitchen to make food. I used to make cucumber salad; I make it for years but no, standing there and make dressing for one person. I don’t bother, and I just buy that ready-made food from the stores.” (P17).

With regard to the domain of personal resources, the participants expressed that a lack of access to personal support was due to families living far away and having almost no contact with relatives or friends. Collectively, the participants stated that access to the senior activity center has a positive effect on maintaining a healthy diet. Furthermore, participants reported a drop in income, expensive healthy food, and transport to be factors in their food choices. Thus, the domain of personal resources was captured using in-depth individual interviews.

“I’ve been here for two years. I’m here because if I am not here, I will be alone in my apartment staring out the window while my husband is at work. I need some social because I’m home, no job, so I have no contact with other people, no siblings, and very little contact with my family.” (P8).

### 4.3. Dyadic Interviews vs. In-Depth Individual Interviews Comparison

The performance of the two interviewing methods is compared in terms of the information elicited (the number of attributes elicited and the attribute variability) and the procedural dimensions (efficiency of data collection and participants’ feedback). 

To address RQ1, we identified the number of attributes that each method elicited. Dyadic interviews generated a higher number of attributes (52 attributes) than the in-depth individual method (37 attributes). The dyadic method yielded data related to both the individual and the collective experience of the two members in the dyad, providing insight into why older adults choose or avoid certain foods. In contrast, the in-depth individual interviews only provided rich data on individuals’ views about what constitutes a healthy diet and how they maintain this in their daily lives.

In addition to eliciting a larger number of attributes, dyadic interviews generated a greater variability of themes. A possible explanation for this might be that more participants were involved in dyadic interviews. This probability, however, is difficult to determine as the equivalent comparison between dyadic and in-depth individual interviews is still unclear [28].

**Figure 1:**
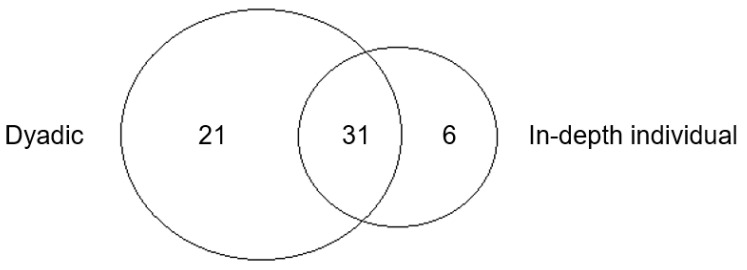
Overlap of attributes retrieved using each method.

In contrast, in-depth individual interviews yielded fewer themes, but more “deep” personal information, such as health conditions, medical procedures, personal economy, and social isolation. This result helps answer RQ2a. Figure 1 shows the extent to which the two methods tap into the specific themes and to what degree the content overlaps. The 52 and 37 attributes elicited in each method, respectively, resulted in a total of 58 different attributes. Of these, 31 emerged in both methods, while 21 were unique to the dyadic method and 6 to the in-depth individual method.

To address RQ2b, we identified the attributes that were captured based on the three domains for the determinants of the food choice of older adults. These are discussed below and summarized in Table 3.

• *Changes Associated with Aging*

The two methods captured age-related changes that influence the food choices of home-living older adults. The in-depth individual interviews yielded a different type of information than dyadic interviews. In the in-depth individual interviews, many of the participants disclosed that physiological changes have affected their past and current food choices. Meanwhile, the data collected from the dyadic interviews concentrated mainly on past challenges, providing little information on the current issues encountered by the participants.

• *Psychosocial Aspects*

Life stage and living arrangement were recognized as the most important social determinants affecting home-living older adults’ food consumption. Our data comparison indicates that the two methods yield generally comparable information. Thus, researchers can use either method to capture the psychosocial aspects.

• *Personal Resources*

Both methods almost fully captured the personal variables that affect older adults’ food choices. These variables are mostly related to the special challenges of maintaining a healthy diet. Participants in the in-depth individual method tend to be more focused on the challenges within themselves, while in the dyadic method, the challenges discussed were based on the participants’ perspectives on understanding other people.

In response to RQ3, we took into account the time spent conducting the interviews, transcribing, and analyzing data. It took between 50 min and 1.5 h to complete a dyadic interview and between 40 min and 1 h to complete an in-depth individual interview.

What follows is the time it took to transcribe and analyze the data. The data collected from the in-depth individual interviews took less time to transcribe and analyze in comparison to the data from the dyadic interviews. Therefore, although there was no significant difference in the amount of time it took to complete interviews for both methods, the data collected from the in-depth individual interviews required much less time to transcribe and analyze.

We will now respond to RQ4 and RQ5. The participants’ reactions to the elicitation method suggest that the dyadic interviews allowed participants to express their opinions with greater ease and to a greater extent than the in-depth individual interviews. It is likely that the broader discussion is a result of the interaction between two people having a shared conversation when responding to interview questions. In contrast, the in-depth individual interviews allowed the participants to open up and share deeper personal feelings. A possible explanation for this might be a reluctance to share their feelings to the fullest extent in the presence of another participant.

Overall, participants found dyadic and in-depth individual interviewing to be a positive experience; however, a few commented on the extensive time investment required by the researchers and the participants to complete the dyadic interviews. In this study, the dyadic interview was clearly not a well-known data collection method among older adults living at home.

### 4.4. Merits and Drawbacks of Using Each Method

In the following section, we will discuss the merits and drawbacks of each method.

#### 4.4.1. Dyadic Interview

As mentioned earlier, it is the interaction in the dyadic interview that produces data [25]. Understanding a pair’s interaction can help us to identify potential merits and drawbacks of the dyadic method, and to further examine these, we include a brief discussion on some pairs’ interactions in our study.

Of the eight pairs of dyads in this study, six pairs shared a pre-existing relationship; thus, establishing pairwise rapport was not a challenge. However, when using the dyadic approach, we have little control over how the participant interaction plays out in the dyads and how the relationship in a dyad may influence the result.

It has been reported that within a dyad, one member of the dyad could dominate by constantly talking and dismissing other opinions [24]. In this study, the evidence of domination can be seen in a married couple and a friend pair. This point is clearly illustrated below.

• *Married Couple*

Of the two married couple dyads we interviewed, domination occurred in one of the couples. Such was the case with Participant 5 (P5), who dismissed his wife’s (P4) statement and continued to cut her off repeatedly, eventually silencing her in the following:

(P5) “I have a strong connection practically and emotionally to local food. For example, the Cider House located in the West. Its cider made from apple and berries, both drinkable and edible.”

(P4) “But cider is made of… (interrupted by P5)

(P5) “Yes yes yes yes yes, they make it from berries as well.”

(P4) “No, they add an extract” (P5 interrupted)

(P5) “Yes yes yes it’s not that but that’s fine”

(P4) “(taking a deep breath), sure”

• *Friend Pair*

In some instances, domination may also operate in a friend pair. Here, one participant (P19) often interrupted and forbade his friend (P18) to disclose more information to the researcher:

(P19) “We are elderly; we don’t need so much food.”

(P18) “I like a big variation, meat and fish, not fatty food and …(interrupted by P19)”

(P19) “Ok, that is enough, I think. Can you just continue with your next question?”

While domination tends to be a concern when conducting dyadic interviews [24], in our case, it provided us with a richer background of how lifestyle and family affected food choices.

What follows is a brief outline of the power relationships that might present between two members of a dyad. It is widely acknowledged that one possible way to neutralize the power dynamic is by asking the participant to identify and invite the person they considered most helpful [12,30]. At the same time, power relationships within families (parent and child) seem inevitable. Given the role that social relationships play in food choices [51], we presume that a dyad between parents and children can provide researchers with richer data.

• *Parent–Child*

It was observed that (P12) seemed to be concerned with her self-image and searched for the “right answer”. Such circumstances can lead to respondent bias, where participants provide socially desirable answers. This can be illustrated briefly by a mother (P12) who is trying to reassure her daughter (P11) that she does not skip her meals, as follows:

(P11) “you eat dinner every day, right, mom (look concerned)?”

(P12) “(look down …sigh) Yes, can’t be full before you eat, so it’s good with dinner.”

(P11) “It is good that you eat dinner; I was worried.”

Lastly, in a setting where two strangers were interviewed together, the participants typically took turns in responding to the interview questions. Throughout the interview, the participants seemed to focus much more on their differences rather than their similarities when answering questions. Although this resulted in a more passive conversation, the stranger’ pairs provided us with a valuable insight into the phenomenon.

When we review all of our dyad pairs, the married couples and friend (female) dyads generated the broadest categories of information. This suggests that pairing a composition with a prior relationship is the more effective form of pairing. Surprisingly, the stranger pairs were found to produce more attributes than the father–son and friend (male) dyad. A likely explanation is that the latter pair of dyads had less interest in the research topic.

Overall, the participants who participated in the homogeneous (two male or two female) or heterogeneous dyad exhibited no meaningful differences. The results show, however, that the female participants had a greater interest in the topic and gained more self-confidence throughout the discussion than the male participants. It can be argued that the positive result for the female participants was due to their higher level of interest in the topic.

#### 4.4.2. In-Depth Individual Interview

The following section discusses the merits and drawbacks of the in-depth individual interview method.

While the relationship between the two participants receives the most attention in dyadic interviews, the researcher–participant relationship is vital in in-depth individual interviews. Researchers using this method tend to establish a closer connection with the participants, which fosters emotions that facilitate a rich interview experience [23]. Hence, a participant’s willingness to share sensitive information is likely to happen in this type of interview.

Furthermore, the participants in the in-depth individual method often took the interview in a different direction [23]. In our case, we followed the participant out of concern and interest. The following is an example:

(P13) “Some circumstances where I eat more than normal, of course, would be if I am at a restaurant and get good food, I eat more of course. So, then you get home, now how much can you… But now Christmas is coming, so now.”

(Researcher): “Yes, any plans for Christmas?”

(P13) “We are going to the diner... the one out there. There will be a lot of ribs and stuff, so then I’ll bring home ribs that I can eat on Christmas Eve because I am alone.”

Although the detailed information can sharpen our ability to understand the complexities of the phenomenon, participants’ privacy and confidentiality must be safeguarded and respected.

More to the point, it is not our intention in this study to probe for sensitive information but to demonstrate the usefulness of this interviewing method and what type of information can be elicited.

It is worth noting that many older adults who participated in the in-depth individual interview experienced loneliness and social isolation. These experiences may have led to a stronger urge to share and confide in others.

## 5. Discussion

Different methods of collecting data from a population of older adults have been used in the literature. However, the suitability of the methods used for this population is rarely studied. In some cases, the pressure to publish could be a reason why researchers tend to choose the most familiar method. In other cases, once the design is matched to the initial research question, the assumption is that the chosen method is flawless [8]. 

In this study, we examined whether the dyadic interview is a suitable method for use with home-living older adults and whether there are differences in eliciting information between a dyadic method and an in-depth individual method in the context of food choice and healthy diets. 

The result of this study shows that more attributes were elicited from the dyadic interviews than the in-depth individual interviews. However, the result also indicated that the content of the two methods overlapped. When we compared the two methods with a specific focus on the type of information that emerged from the interviews, the in-depth individual interviews revealed more personal and sensitive data. In contrast, the dyadic interviews covered a broader area related to the topic of food and healthy diets. The results thus support the findings reported in a previous study [9]. 

While the study by Morgan, Ataie, Carder and Hoffman [9] was conducted to illustrate some methodological aspects of the dyadic interview, our study extends these findings by systematically comparing dyadic interviews with in-depth individual interviews. Contrary to findings in previous research [28], this study reveals that recruiting participants for dyadic interviews took as much time as recruiting participants for in-depth individual interviews. This rather contradictory result may be due to the gatekeeper permission required to access participants and the unique characteristics of an older population. However, it is worth noting that it took considerably longer to transcribe and analyze the dyadic interviews than the in-depth individual interviews. 

When evaluating the participants’ reactions to the interview methods, both methods yielded positive feedback. The fact that participants expressed positive feelings suggests that the dyadic interview is a viable method for collecting qualitative data from a sample of older adults.

The findings also highlight the importance of participant selection when pairing a dyad, an area of inquiry that has been relatively under-studied in the qualitative literature. By pairing participants based on different relationships, we provide insight into selecting a dyad pair.

Regarding knowledge production, we initially thought that people would merely share information about their own food choices in the in-depth individual interviews. It turns out that the participants occasionally represent themselves and their partners even though they were interviewed alone. As such, the approach allows one voice to represent two people. In contrast, the dyadic interviews allow two participants to present their perspectives individually alongside the dyadic perspective created by the participants together. Thus, dyadic interviews open up for more voices to be heard in knowledge production [10].

Having applied the two interview methods, this study improved our understanding of choosing the “right” tools for data collection that enable researchers to find answers to research problems.

To the best of our knowledge, this is the first study that explicitly and empirically compares dyadic interviews and in-depth individual interviews using home-living older adults as a sample. Our findings suggest that the dyadic interview is an appropriate method for collecting data from home-living older adults. Therefore, we would encourage researchers in qualitative studies to adopt the dyadic method when interviewing older adults in a food-related context and beyond.

## 6. Limitations and Future Direction

Our research design enables us to evaluate two different methods of eliciting factors in food choice domains and to examine what information can be captured through deliberate heterogeneous sampling. Thus, this study went beyond simple method comparison to the higher research design level. 

This being said, this study was an attempt to expand our understanding of data collection methods in qualitative research. The limitations of the study, which included sampling and inherent methodological issues, must be delineated. 

The study involved only a small sample of older adults in one region of Norway who were native Norwegian. As such, the result of the study is limited to the selected participants and their experience related to food choices. To reduce this challenge, we applied deliberate sampling for heterogeneity. Deliberate sampling for heterogeneity is recommended as the best alternative when random sampling cannot be used [52,53]. As a result, it allowed us to look at sample members from all available angles, thereby achieving depth understanding of the phenomenon.

In addition, the setting, and the cultural and societal differences of the study may also limit the transferability of the results to other contexts and situations beyond the scope of this study. Therefore, the findings presented in this article should be interpreted with caution.

What follows is the influence of researcher bias. We sought to reduce these biases by actively thinking reflexively throughout the research process and adopting different types of triangulation (method triangulation, investigator triangulation, and data source triangulation).

Moreover, in terms of sample size, our intention was to have an adequate sample size. However, the adequate sample size needed for qualitative research findings to have some validity is difficult to estimate [54]. One way to increase the validity of our findings would have been to increase the number of participants to reach theoretical saturation [55]. In the case of the dyadic interviews, this would have involved arranging for more pairs to be interviewed. 

However, due to limited resources and time, we chose to sample heterogeneity instead of increasing the sample size. We postulated that such sampling would yield a sufficient breadth and depth of the phenomenon being studied. 

In spite of these limitations, this study does provide insights that can guide future exploration of the dyadic method. This study is a good starting point, but far more empirical research is needed on the dyadic method. Finally, we used food choice as a context in the present study. Further research in a different context is recommended as a means to determine the efficacy and efficiency of the dyadic method.

## Figures and Tables

**Table 1:** Description of participant characteristics from dyadic interviews.

Dyad Pairs	Gender	Age	Occupation	Employment Status	Marital Status	Living Situation
**Married couple 1**	Female	64	Teacher & counselor	Full-time	Married	Living with a spouse
	Male	66	Engineer	Retired		
**Married couple 2**	Female	62	Manager at kindergarten	Part-time	Married	Living with a spouse
	Male	65	Substance abuse-related psychiatrist	Full-time		
**Father-Son**	Male	60	Counselor in an office	Full-time	Divorced	Living alone
	Male	28	Teacher	Full-time	In a relationship	Living with a partner
**Mother-Daughter**	Female	86	Shopkeeper	Retired	Widow	Living alone
	Female	58	Teacher	Full-time	Divorced	Living alone
**Friends pair 1**	Female	88	Tour guide	Retired	Widow	Living alone
	Female	83	Travel agency	Retired	Widow	Living alone
**Friends pair 2**	Male	80	Engineer	Retired	Married	Living with a spouse
	Male	76	Engineer	Retired	Divorced	Living alone
**Strangers pair 1**	Male	72	Civil engineer	Retired	Married	Living with a spous
	Male	69	Engineer	Retired	Single	Living alone
**Strangers pair 2**	Female	63	Housewife	Unemployed	Married	Living with a spouse
	Male	60	Teacher	Full-time	Married	Living with a spouse

**Table 2:** Description of participant characteristics from in-depth individual interviews.

Participants	Gender	Age	Occupation	Employment Status	Marital Status	Living Situation
**Participant 3**	Female	82	Housewife	Unemployed	Widow	Alone
**Participant 8**	Female	58	Housewife	Unemployed	Married	With spouse
**Participant 13**	Female	92	Housekeeper	Retired	Widow	Alone
**Participant 16**	Male	88	Businessman	Retired	Widow	Alone
**Participant 17**	Male	71	Skipper	Retired	Widow	Alone
**Participant 20**	Male	71	Petroleum engineer	Retired	Single	Alone

**Table 3:** Factors contributing to or impeding the food choices of older adults living at home.

Domains of Food Choice		Dyadic Interviews	In-Depth Individual Interviews
Changes associated with aging	Taste		x
	Poor dentition		x
	Loss of appetite	x	x
	Mobility or functional limitations	x	x
	Illness or medical conditions	x	x
Psychosocial aspects	Life-course	x	
	Living arrangement	x	
	Self-perception of health status	x	x
	Desire for independence	x	
	Lack of motivation or energy	x	x
	Personal interest in health/nutrition	x	
Personal resources	Transportation issues		x
	Income/food costs	x	x
	Access to personal support	x	x
	Knowledge/skills in food preparation	x	x
	Access to quality products	x	x
	Dietary resilience to overcome barriers encountered	x	

## Data Availability

The data presented in this study are available on request from the corresponding author. The data are not publicly available due to privacy and ethical restrictions.
